# Investigation of *Fasciola gigantica* in freshwater snail *Radix* (*Lymnaea*) spp. In the highly parasite-prevalent area of Nakhon Ratchasima Province, Thailand

**DOI:** 10.1080/23144599.2024.2396700

**Published:** 2024-09-10

**Authors:** Pongsakorn Martviset, Pathanin Chantree, Amornrat Geadkaew-Krenc, Pantip Piyatadsananon, Ruttiroj Jirojwong, Chompunoot Wangboon, Mantana Jamklang, Sirilak Chumkiew, Rawipreeya Poomkhokrak, Nanthawat Kosa, Salisa Chaimon, Bumpenporn Sanannam, Rudi Grams, Wansika Phadungsil

**Affiliations:** a Department of Preclinical Science, Faculty of Medicine, Thammasat University, Pathum Thani, Thailand; b Graduate Program in Applied Biosciences, Faculty of Medicine, Thammasat University, Pathum Thani, Thailand; c Graduate Studies in Biomedical Sciences, Faculty of Allied Health Sciences, Thammasat University, Pathum Thani, Thailand; d Institute of Science, Suranaree University of Technology, Nakhon-Ratchasima, Thailand; e Department of Livestock Development, Ministry of Agricultural and Cooperatives, Nakhon-Ratchasima, Thailand; f Suranaree University of Technology Hospital, Nakhon-Ratchasima, Thailand

**Keywords:** *Fasciola gigantica*, *Lymnaea (Radix)* spp, freshwater snail, Thailand

## Abstract

This study investigates the distribution of the *Lymnaea* (*Radix*) spp. in Pak Chong district, Nakhon Ratchasima province, northeast Thailand, where a vast cattle farming area is located and has a high prevalence of *Fasciola* spp. in the cattle. By random selection, 1,414 snails were collected from the natural and man-made ponds. The snails were recorded for morphology and processed for DNA isolation. The snail species were investigated by conventional PCR using a 16S rDNA-specific primer. The result demonstrated that all collected snails were *R*. (*L*.) *rubiginosa*. Moreover, the infection of *Fasciola* gigantica in the snails was investigated by PCR using a cytochrome c oxidase I (COX1)-specific primer. The results illustrated that the overall prevalence was 22.5% (318/1414), with the highest prevalence in the Nong Sa Rai subdistrict at 73.6% (81/110), which is the highest prevalence of *Fasciola gigantica* in the snail host that has ever been reported. The lowest prevalence existed in the Pong Ta Long subdistrict at 3.7% (4/109). Our results corresponded to the previous report on the *Fasciola* spp. infection in the cattle from this area, and the geographical analysis revealed that the most suspected factor would be the earth dam located in these subdistricts, where many animals live freely during the day. Our findings could be helpful for further parasite control and could trigger the study of the biology and associated factors in the future.

## 1. Background

Parasitic infection is a significant health problem for animals worldwide, specifically, economically important animals such as cattle, including beef and dairy cattle [1,2]. The infection not only causes undesirable conditions that affect animal health but could also reduce the quantity and quality of animal products, including meat, milk, wool, etc [1]. Moreover, the infection sometimes causes death and can be transmitted to humans as a zoonotic disease [2,3]. In fact, the parasites could be nematodes, trematodes, cestodes, or unicellular parasites, which cause diverse complications [4].

Thailand is a tropical country located in the Southeast Asia (SEA) where the agricultural industry is a huge economic machinery, and the cattle are in that government’s promotion. In 2021, the cattle population in Thailand was probably more than 8.3 million, and the northeastern region is the biggest farming area [5]. Nakhon Ratchasima Province, the largest province of Thailand in terms of area size, is located in the northeastern region and serves as the biggest cattle farming area, with more than 581 thousand registered cattle in 2021 [5].

Our previous study reported the high copro- and seroprevalence of *Fasciola* spp. in dairy and beef cattle in the Pak Chong district of Nakhon Ratchasima province, which is a huge farming area in Thailand [6]. *Fasciola* spp. comprise two well-known species, *Fasciola hepatica* and *Fasciola gigantica*, which cause fasciolosis or liver rot disease [7–9]. They have been reported in all continents, especially South America, the Middle East, Southeast Asia, and Oceania [7]. It is estimated that more than 700 million animals and about 2.4 million people worldwide are infected with such disease [8,10–12]. The pathogenesis of this disease varies, ranging from asymptomatic to severe symptoms, definitely hepatic fasciolosis [7,8,13]. The most affected animals include sheep, goats, and cattle [14]. As mentioned, fasciolosis also causes economic losses, with a share of more than 3.2 million USD in approximately [3,7,15,16]. The parasite life cycle is well-studied that starts when the host eats infective metacercaria. The metacercaria excysts in the small intestine, then the newly excysted juvenile (NEJ) penetrates the intestinal wall, travels through the peritoneal cavity, develops into an adult in the liver parenchyma, and stays in the large biliary tracts causing various symptoms [8,11,13,17–20]. The adult parasite produces unembryonated eggs and then passes to the environment via faeces. The embryo inside the eggs develops to miracidium, hatches, and infects the freshwater snails, specifically lymnaeid snails, by penetration. The lymnaeid snail is a small to large air-breathing freshwater snail in freshwater ponds. It is an aquatic pulmonate gastropod mollusc in the subfamily Lymnaeinae of the family Lymnaeidae, which includes numerous species that serve as intermediate hosts for trematodes [21,22]. Inside the snail, the parasite produces numerous sporocysts which transform into redia, which later transform into cercaria. The cercaria can freely swim out from the snail host and become encysted and attached to the water plant called metacercaria, which is an infective stage [8]. For this reason, in the case of *Fasciola* spp., the snail intermediate host is very important as it could be a dump to produce a lot of cercaria, which later become metacercaria [18].

Hence, this study aims to investigate the infection rate in the snail intermediate host of *Fasciola* spp., *Lymneae* (*Radix*) spp., in the Pak Chong district of Nakhon Ratchasima province in the northeast of Thailand, where this parasite is highly prevalent. This area, which is crucial for cattle farming, has never been investigated before. For high sensitivity, we used the molecular detection method both to identify snail species and to detect *Fasciola* gigantica. The results of this study will improve *Fasciola* gigantica infection control in the future.

## 2. Materials and methods

### 2.1. Ethical statement and biosafety approval

This study was ethically approved by the Thammasat University Animal Care and Use Committee (protocol no. 007/2022), and all procedures were performed in accordance with the recommendations in the Guide for the Care and Use of Laboratory Animals of the National Institute of Health. The laboratory procedures concerning biosafety were accredited by the Thammasat University Institutional Biosafety Committee (reference no. 042/2565).

### 2.2. Study areas, sample size calculation, snail sample collection, and process

**Figure 1.**
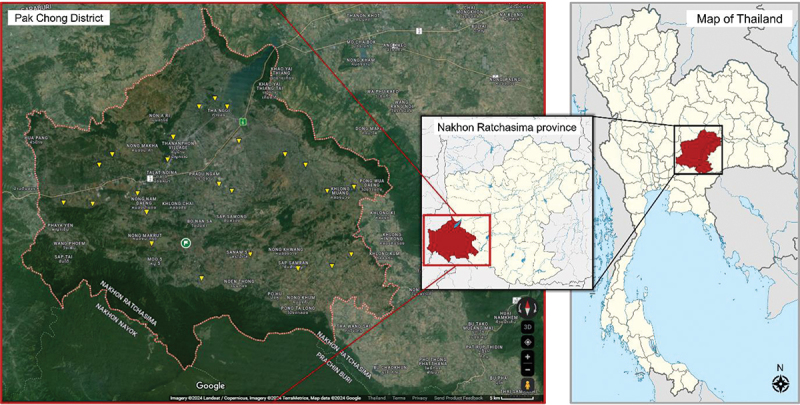
Map of Pak Chong district, Nakhon Ratchasima province, the study site (Map from https://commons.m.wikimedia.org/wiki/Atlas_of_Thailand). The yellow spots indicate the snail’s collected ponds.

This study was performed in the Pak Chong district of Nakhon Ratchasima province, northeast Thailand (Figure 1), which is a vast farming area of cattle, sheep, and goats with more than 90 thousand registered animals in 2022 [5]. A cross-sectional study was conducted between October 2022 and May 2023. The sample size was calculated using a single-proportion formula [23] as follows:

$$n=\frac{Z^{2}\left(1-\frac{\propto }{2}\right)P\left(1-P\right)}{d^{2}}$$

The parameters included: n was the sample size, Z was the statistics corresponding to a 95% confidence interval (1.96), α was 0.05, d was the precision of estimation (0.05), and P was the assumed prevalence of *Fasciola* spp. in this area (according to the previous study; 0.24 [6]). The adjusted calculated sample size was 272 samples.

For the sample collection, twenty-four natural ponds and man-made reservoirs were randomly selected from all twelve subdistricts of Pak Chong district (as marked in Figure 1). Landing nets were used to collect snails in the early morning from 6 to 8 a.m. or around sunset from 5–7 p.m. At each reservoir, five people spent approximately one hour each collecting snails during each collection period. Prior to capture, each snail was observed for key morphological features of *Radix* (*Lymnaea*) spp., including shell characteristics, whorl pattern, and ear-shaped aperture, as previously described [24]. A total of 1,414 snails were collected and included in this study, which was higher than the sample size calculated for greater accuracy. The collected snails were recorded for their external characteristics, snap-freeze, and kept at −20°C until used for DNA isolation.

### 2.3. DNA isolation

The E.Z.N.A.® Mollusc DNA Kit (Omega Bio-Tek, Norcross, GA, USA) was used for the extraction of snail DNA. Briefly, the snail was removed from the shell using clean forceps, and 30 mg of snail tissue was excised and used for DNA isolation. The snail tissue was cut into small pieces and incubated in 350 µl ML lysis buffer containing 25 µl proteinase K solution at 60°C for 2 hours until the entire sample was solubilized. The undesirable proteins in the lysate were extracted by phenol/chloroform, and the RNAs were removed by incubating with 10 µl RNase in 350 µl BL Buffer at 70°C for 10 minutes. The DNA was precipitated using 100% ethanol and loaded into a HiBind® DNA Mini Column. The column was centrifuged at 10,000*×g* for 1 min and washed with 700 µl wash buffer twice. The DNA was eluted from the column by 50 µl elution buffer and harvested by centrifugation at 10,000×g for 1 min. The concentration of isolated DNA was measured using the nanodrop spectrophotometer (NanoDrop One, Thermo Fisher Scientific, Wilmington, MC, USA) and stored at − 20°C until used.

### 2.4. Polymerase chain reaction (PCR) amplification

The 1,414 individual snails were molecularly identified by PCR amplification using the primer designed from *Radix* 16S rDNA (GenBank accession no. GU167907.1) modified from previously reported [25]. The sequence of forward primer was 5´-TTT TAA TAA AGA ATT TTC TGT CTT CTT TAA A-3´ and reverse primer was 5´-TCA CAA AAC TTA ATG TCC AGT GGA GTA TTT-3´. The PCR reaction was carried out in the thermocycler using GoTaq® Colorless Master Mix (Promega, Madison, WI, USA). The 25 µl reaction mixture included 12.5 µl 2X GoTaq® Master Mix, 25 pMol of each primer (forward and reverse primers) and 500 µg/ml of DNA template. The amplification reactions consisted of initial denaturation at 95°C for 5 min, followed by 35 cycles of 95°C for 1 min, 55°C for 1 min, and 72°C for 1 min. The final extension was done at 72°C for 10 min.

For the prevalence of *F. gigantica*, we used the *F. gigantica* COX1 gene (GenBank accession no. NC_024025.1) as a detection target [26,27]. The forward primer sequence was 5´-TGA CGG GGC ATG GTG TTA TT-3´, and the reverse primer sequence was 5´-CAG TAC CCT CGC CCA ACA TA-3´. The genomic DNA of adult *F. gigantica* was used as a positive control. The PCR amplification reaction was performed in the same condition as *Radix* 16S rDNA and the PCR amplicons were resolved on 1% agarose gels containing ViSafe Green Gel Stain (Vivantis Technologies, Selangor, Malaysia) and visualized under Blue light (UVCI-1100. Major Science, Taoyuan, Taiwan).

### 2.5. Purification of PCR products and DNA sequencing

The pattern of the PCR amplicons was observed, and one sample from each subdistrict was selected and subjected to verify by DNA sequencing. The PCR products were purified using a GF-1 PCR clean-up kit (Vivantis Technologies, Selangor Darul Ehsan, Malaysia). The concentration of purified PCR products was measured using a NanoDrop™ spectrophotometer (NanoDrop One, Thermo Fisher Scientific, Wilmington, MC, USA). The DNA sequences were identified by Sanger sequencing using the service of Solgent Co. Ltd. (Daejeon, South Korea). The sequencing results were verified using the nucleotide BLAST tool on GenBank (https://blast.ncbi.nlm.nih.gov/) [28].

### 2.6. Phylogenetic analyses

To investigate the genetic diversity of snails and parasites, the twelve DNA sequences of each *Radix*16S rDNA (192 bp) and of *Fg*COX1 (351 bp) were multiple-aligned using CLUSTAL W version 2.1 [29]. As mentioned above, the analyses were carried out based on the previously published sequence of *Radix* 16S rDNA and *F. gigantica* COX1. The phylogenetic tree was generated by MEGA11 [30] using the maximum likelihood method with 1000 bootstrap replications.

### 2.7. Geographic localization of parasitic hotspot

The geographic localization was performed after getting the prevalence data by using the locations of the water reservoirs that obtained the snail samples. The geographic information system (GIS) data were recorded using Google Maps and a handheld global positioning system (GPS) (Garmin eTrex 32×, Lenexa, KS, USA), including the longitude and latitude of the water reservoirs with experimental uncertainty of ±5 metres. The obtained locations were imported to ArcGIS desktop software and analysed using ArcMap version 10.3 (Environmental Systems Research Institute, Redlands, CA, USA). Cluster analysis was performed to evaluate the relationship between the geographic findings and parasite-positive reservoir locations by spatial autocorrelation using Global Moran’s I. A *p-*value less than 0.05 was considered statistically significant.

## 3. Results

### 3.1. Macroscopic morphology of collected Lymnaeid snails

**Figure 2.**
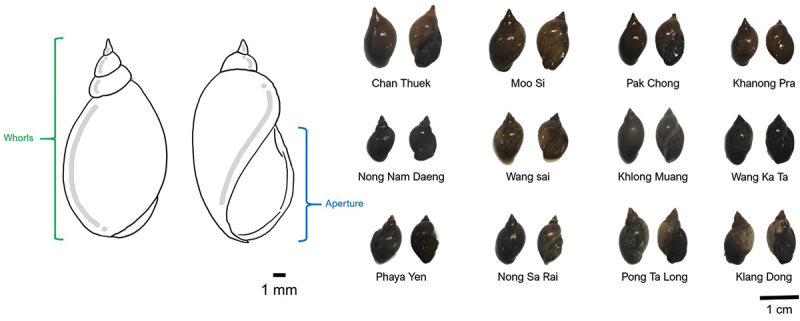
The morphological appearance of Lymnaeid snails collected from each subdistrict of Pak Chong district, Nakhon Ratchasima province, Thailand.

All collected snails have a similar external appearance related to *Lymnaea* spp., as previously mentioned [24]. The sizes varied, ranging from 0.9 to 2.0 cm in length and 0.3 to 0.7 cm in width. The shell colours were light brown to dark brown; some contained light spots all over the shell, and dark stripes could be observed in some collected snails. They lacked an operculum; the opening aperture was on the right when facing towards, and the spire was pointing upwards. The outer edge was S-shaped, and the columella was distorted and folded (Figure 2), which was a characteristic of *Lymnaea* (*Radix*) sp.

### 3.2. Genetic diversity of collected Lymnaeid snails

**Figure 3.**
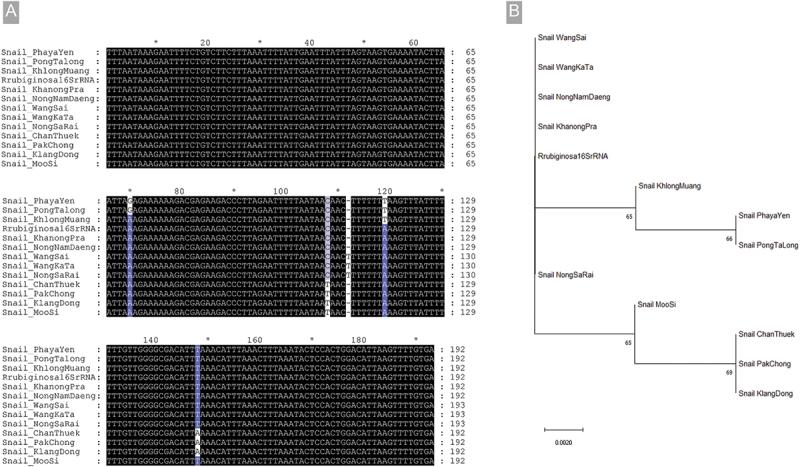
(A) Multiple alignments of sequenced PCR amplicons (192 bp) of *Radix* 16S rDNA-specific primer from snail DNA samples of each subdistrict. The highlighted bases indicate the base alteration found in the sequences compared to the reference sequence (*Rrubiginosa*16SrRNA). (B) Phylogenetic analysis of the snails collected from each subdistrict of Pak Chong district, Nakhon Ratchasima province.

The PCR amplification of *Radix* 16S rDNA-specific primer using snail DNA was done in all samples. The amplification pattern was identified; fortunately, all samples showed the same band pattern, so one sample from each subdistrict was sent for DNA sequencing. The PCR amplicons from all subdistricts were in the expected size (192 bp), as shown in Figure S1. After sequencing, the results of Blastn showed that all sequences had high similarity, ranging from 98.4 to 100% with *R. (L.) rubiginosa* reference sequence, suggesting its identification as this species. Interestingly, the sequence details of some snail samples illustrated a little difference. The snails from Khanong Pra and Nong Nam Daeng subdistricts demonstrated 100% conservation to the reference sequence (GenBank accession no. GU167907). The snails from Wang Sai, Wang Ka Ta, and Nong Sa Rai also demonstrated 100% conservation, but there was one additional base at the base no. 112. On the other hand, the snails from Moo Si had one different base at base no.108 that changed from C to T. The base change at this location could also be observed in the snails collected from Chan Thuek, Pak Chong, and Klang Dong subdistricts, but they had more alteration at base no. 147, which changed from T to A. The change in the base was also observed in the snails collected from Phaya Yen, Pong Ta Long, and Khlong Muang at base no. 119, which was changed from A to T. Moreover, Phaya Yen and Pong Ta Long presented another alteration at base no. 90. The multiple alignments demonstrated all the differences compared with the reference sequence, as shown in Figure 3A. These findings also illustrated in the phylogenetic analyses that the snails from Wang Sai, Wang Ka Ta, Nong Nam Daeng, and Khanong Pra were clustered with *R. rubiginosa*. The snail from Nong Sa Rai separated into its own branches. The snails from Khlong Muang, Phaya Yen, and Pong Ta Long were clustered in one major branch, and Moo Si, Chan Thuek, Pak Chong, and Klang Dong were grouped in another cluster. All sequences of the snails obtained from our study were submitted to the GenBank database, and the sequences and accession numbers are shown in Table S1.

### 3.3. Prevalence of *Fasciola* gigantica In the snails

The molecular prevalence was done by conventional PCR using *Fasciola* gigantica COX1-specific primer. The positive samples demonstrated the PCR amplicons at the expected size of 351 bp, as shown in Figure S2. The overall prevalence was 22.5% (318/1414). The highest prevalence was found in Nong Sa Rai subdistrict at 73.6% (81/110), followed by Chan Thuek at 43.7% (52/119), Pak Chong at 40.3% (50/124), Nong Nam Daeng at 22.0% (28/127), Khanong Pra at 21.6% (27/125), Wang Ka Ta at 13.9% (16/115), Phaya Yen at 13.0% (14/108), Khlong Muang at 11.8% (13/110), Moo Si at 10.8% (13/120), Klang Dong at 8.2% (9/110), Wang Sai at 8.0% (11/137), and the lowest prevalence was observed in Pong Ta Long at 3.7 (4/109), respectively. The prevalence of all collected snail samples is represented in Table 1.

**Table 1.** The prevalence of *Fasciola gigantica* in Lymnaeid snails collected from each subdistrict of Pak Chong district, Nakhon Ratchasima province, Thailand.

Subdistrict	No. of snail samples	Prevalence (%)
Khanong Pra	125	21.6 (27/125)
Nong Nam Daeng	127	22.0 (28/127)
Chan Thuek	119	43.7 (52/119)
Pak Chong	124	40.3 (50/124)
Klang Dong	110	8.2 (9/110)
Phaya Yen	108	13.0 (14/108)
Pong Ta Long	109	3.7 (4/109)
Klong Muang	110	11.8 (13/110)
Wang Sai	137	8.0 (11/137)
Wang Ka Ta	115	13.9 (16/115)
Nong Sa Rai	110	**73.6 (81/110)**
Moo Si	120	10.8 (13/120)
**Overall**	**1414**	**22.5 (318/1414)**

### 3.4. Genetic diversity of *F.* *gigantica*

**Figure 4.**
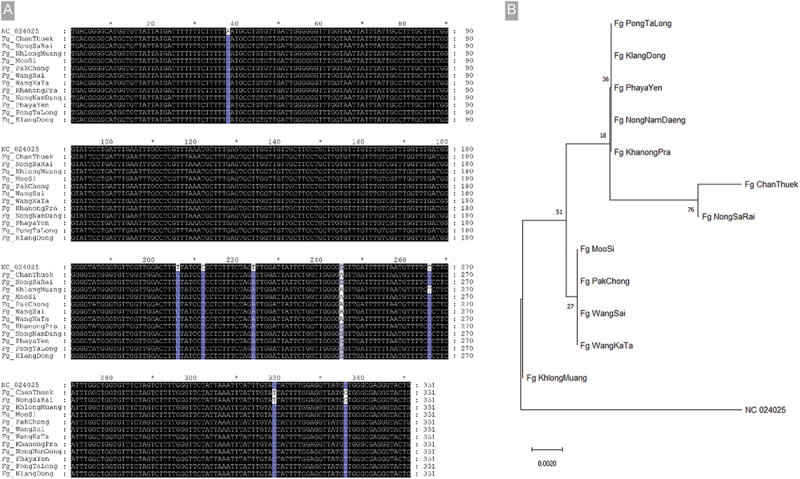
(A) Multiple alignment of sequenced PCR amplicons of *F. gigantica* COX1-specific primers (351 bp) from snail DNA samples from each subdistrict. The highlighted bases indicate the base alteration found in the sequences compared to the reference sequence. (B) Phylogenetic analysis of the *F. gigantica* from the collected snails from each subdistrict of Pak Chong district, Nakhon Ratchasima province.

The multiple alignment of sequencing results of PCR amplicons from *F. gigantica* COX1-specific primers were interesting when compared to the reference sequence. Surprisingly, no sequence from any samples showed 100% similarity to the *F. gigantica* reference. The most closely related was collected from the Khlong Muang subdistrict, which has an alteration of four bases where all collected samples had the same pattern but differed from the reference sequence. All sequences were totally different from the reference at base no. 38 (A to G), 206 (T to C), 212 (C to T), and 224 (T to A). Interestingly, the other sequences obtained from the subdistricts had base alterations at different locations, as shown in Figure 4A. However, the proportions of similarity remained high, ranging from 97.7% to 98.5%. The phylogeny demonstrated that *F. gigantica* from the Khong Muang subdistrict was the most relevant. The sequences from Moo Si, Pak Chong, Wang Sai, and Wang Ka Ta were clustered in one relative branch, Pong Ta Long, Klang Dong, Phaya Yen, Nong Nam Daeng, and Khanong Pra were clustered in another relative branch, and Chan Theuk and Nong Sa Rai were grouped furthest away from the reference (Figure 4B). All sequences of *F. gigantica* obtained from our study were submitted to the GenBank database, where accession numbers are shown in Table S2.

### 3.5. Geographic analysis of the infected snails

**Figure 5.**
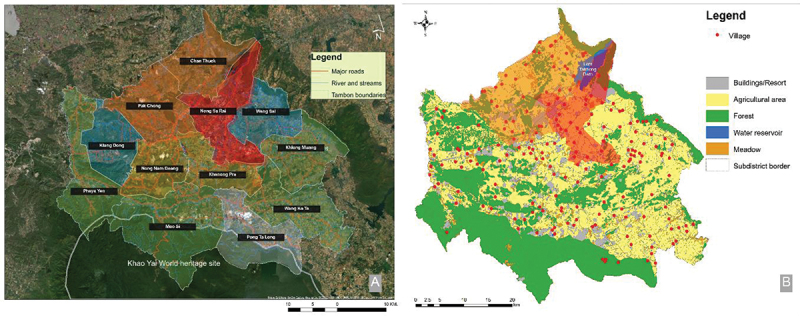
Geographical analysis of the infected snails in each subdistrict of Pak Chong district, Nakhon Ratchasima province. The coloured subdistricts represent the variation of the prevalence, including red, which has a prevalence of more than 70%; orange, 40-70%; yellow, 20-30%; green, 10-20%; blue, 5-10%; and lower than 5% is labelled in white.

The prevalence data was used to analyse the geographical distribution of the parasite in the snail host, as illustrated in Figure 5. The result demonstrated that the highest prevalence subdistrict, Nong Sa Rai subdistrict (labelled in red), is closely related to the considerable earth dam (Lam Takhong dam). Moreover, the second and third highest prevalent subdistricts (labelled in orange) were also close to the dam. The subdistricts that had a lower prevalence (labelled in yellow for the prevalence of 20–30%, green for 10–20%, and blue for 5–10%) were located far away from the dam. Noteworthy, the lowest prevalent subdistrict, Pong Ta Long subdistrict (labelled in white), is located in the mountain zone very far from the dam.

## 4. Discussion

Fasciolosis, a parasitic infectious disease, is one of the neglected tropical diseases that causes undesirable conditions in humans and animals worldwide [3,14]. Drug administration in the infected organisms, as well as controlling the snail intermediate host, could be helpful for parasite control. The snail intermediate host is crucial because it amplifies the developmental stages of the parasite, which finally develops into metacercaria, which is an infective stage [8]. So, studying the infectivity of the specific snail host of the parasite is crucial for further control.

Our previous study reported that the prevalence of *Fasciola* spp. in cattle in one of Thailand’s colossal farming areas, Pak Chong Highland, was very high, especially in beef cattle, where seroprevalence reached 45.5%, and coproprevalence was up to 11.1% [6]. That study indicated that the beef cattle mostly consumed natural grasses and drank natural water, which inspired us to investigate the infection in the snail host, which is the intermediate host of the parasite. In this present study, 1,414 Lymneiad snails were randomly collected from twenty-four natural and man-made ponds located in the Pak Chong district of Nakhon Ratchasima province. All collected snails were externally and molecularly identified as *R. (L.) rubiginosa*. This result suggested that our finding corresponds to the previous reports that *R*. (*L*.) *rubiginosa* is the major *Lymnaea* species in Thailand that is distributed all over the country [25,31–34]. However, our study demonstrated that there are some genetic differences between the snails collected from different locations, but they remain in the same species. Moreover, our study found that the different genetic diversity does not affect the infection rate of the parasite in the snails.

Interestingly, our study found that one of the subdistricts (Nong Sa Rai subdistrict) of Pak Chong district had an extremely high prevalence of *F. gigantica*, reaching 73.6%. Additionally, not only the Nong Sa Rai subdistrict but also the other two subdistricts have a prevalence of more than 40%, and the other two have more than 20%. This finding suggests that the animals and humans living in this area are at risk of infection with *F. gigantica* from eating the water plants containing metacercaria, drinking contaminated water from the natural pond, or accidentally when swimming in the ponds. When compared to the other studies, our study demonstrated a higher percentage than the studies in South Africa (46%), China (overall prevalence of 9.7%, with the highest in the plateau area of 36.9%) [35], Turkiye (1.3%) [36], and India (6.2%) [27].

Correspondingly, the Nong Sa Rai subdistrict also had the highest prevalence in beef and dairy cattle, both copro- and seroprevalence in the previous report [6]. When coupled with the geographical analysis in this study, the hotspots will be located near the Lam Takhong Dam (the massive earth dam) in Nong Sa Rai and Chan Thuck subdistricts (the second most prevalent subdistrict). It is very reasonable because the beef cattle that are farmed in this area are mainly in an open system that allows them to be fed with natural grasses and water from the dam and branched canals [6]. The elements are ideally provided, including infected animals, water reservoirs, snail hosts, and water plants, making a better chance for the completion of the parasite life cycle. This information is very important for the farmers in Thailand, especially in this area of study, who need interventions to control not only the parasites in the infected animals but also the infective snail control, which is crucially needed to stop the endemic.

Additionally, the genetic alteration of the parasite found in the snails is very attractive. The molecular analysis demonstrated that all of the *F. gigantica* found in our study were genetically different from the reference strain. It is very interesting because hybrid species are increasingly reported worldwide [9,37].

In conclusion, we reported high numbers of *F. gigantica* —infected *R*. (*L*.) *rubiginosa* snails in Thailand’s colossal farming area. Our findings suggested that the animals and humans who live in this area are at risk of parasitic infection and should avoid using natural water, and it alerts the veterinarians and healthcare workers in this area to take action on this situation. Moreover, our study confirmed that the snail intermediate host contributes to the high prevalence of *F. gigantica* in the animal farms in this area, as previously reported. Our finding is beneficial for parasite control policy, not only in this country, but also in other regions of the world where *Fasciola* spp. is endemic.

## Supplementary Material

Supplementary Tables revised 2.docx

Supplementary Figures revised 2.docx

## Data Availability

Data is available on request due to privacy/ethical restrictions.
