# Supplementary material for: Investigation of Fasciola gigantica in freshwater snail Radix (Lymnaea) spp. In the highly parasite-prevalent area of Nakhon Ratchasima Province, Thailand
Source: Int J Vet Sci Med. 2024 Sep 10;12(1):125–33. doi: 10.1080/23144599.2024.2396700 (PMC11389629; doi:10.1080/23144599.2024.2396700)
Supplement: Supplementary Tables revised 2.docx [file TVSM_A_2396700_SM2853.docx]

**Supplementary Table**

**Table S1** All of the snail 16S rDNA sequences obtained from our study with their accession numbers.

| **Name code** | **GenBank accession no. and sequence** |
| --- | --- |
| Snail_ChanThuek | >PP913359 Rr16SrRNAChanthuek [organism= Radix rubiginosa] Radix rubiginosa isolate 16S ribosomal RNA gene, partial sequence  TTTAATAAAGAATTTTCTGTCTTCTTTAAATTTTATTGAATTTATTTAGTAAGTGAAAATACTTAATTAAAGAAAAAAGACGAGAAGACCCTTAGAATTTTTAATAATAACTTTTTTAAAGTTTATTTTTTTGTTGGGGCGACATTAAAACATTTAAACTTTAAATACTCCACTGGACATTAAGTTTTGTGA |
| Snail_MooSi | >PP913360 Rr16SrRNAMuSi [organism= Radix rubiginosa] Radix rubiginosa isolate 16S ribosomal RNA gene, partial sequence  TTTAATAAAGAATTTTCTGTCTTCTTTAAATTTTATTGAATTTATTTAGTAAGTGAAAATACTTAATTAAAGAAAAAAGACGAGAAGACCCTTAGAATTTTTAATAATAACTTTTTTAAAGTTTATTTTTTTGTTGGGGCGACATTTAAACATTTAAACTTTAAATACTCCACTGGACATTAAGTTTTGTGA |
| Snail_PakChong | >PP913361 Rr16SrRNAPakChong [organism= Radix rubiginosa] Radix rubiginosa isolate 16S ribosomal RNA gene, partial sequence  TTTAATAAAGAATTTTCTGTCTTCTTTAAATTTTATTGAATTTATTTAGTAAGTGAAAATACTTAATTAAAGAAAAAAGACGAGAAGACCCTTAGAATTTTTAATAATAACTTTTTTAAAGTTTATTTTTTTGTTGGGGCGACATTAAAACATTTAAACTTTAAATACTCCACTGGACATTAAGTTTTGTGA |
| Snail_KhanongPra | >PP913362 Rr16SrRNAKhanongPhra [organism= Radix rubiginosa] Radix rubiginosa isolate 16S ribosomal RNA gene, partial sequence  TTTAATAAAGAATTTTCTGTCTTCTTTAAATTTTATTGAATTTATTTAGTAAGTGAAAATACTTAATTAAAGAAAAAAGACGAGAAGACCCTTAGAATTTTTAATAACAACTTTTTTAAAGTTTATTTTTTTGTTGGGGCGACATTTAAACATTTAAACTTTAAATACTCCACTGGACATTAAGTTTTGTGA |
| Snail_NongNamDaeng | >PP913363 Rr16SrRNANongNamDaeng [organism= Radix rubiginosa] Radix rubiginosa isolate 16S ribosomal RNA gene, partial sequence  TTTAATAAAGAATTTTCTGTCTTCTTTAAATTTTATTGAATTTATTTAGTAAGTGAAAATACTTAATTAAAGAAAAAAGACGAGAAGACCCTTAGAATTTTTAATAACAACTTTTTTAAAGTTTATTTTTTTGTTGGGGCGACATTTAAACATTTAAACTTTAAATACTCCACTGGACATTAAGTTTTGTGA |
| Snail_WangSai | >PP913364 Rr16SrRNAWangSai [organism= Radix rubiginosa] Radix rubiginosa isolate 16S ribosomal RNA gene, partial sequence  TTTAATAAAGAATTTTCTGTCTTCTTTAAATTTTATTGAATTTATTTAGTAAGTGAAAATACTTAATTAAAGAAAAAAGACGAGAAGACCCTTAGAATTTTTAATAACAACTTTTTTTAAAGTTTATTTTTTTGTTGGGGCGACATTTAAACATTTAAACTTTAAATACTCCACTGGACATTAAGTTTTGTGA |
| Snail_KhlongMuang | >PP913365 Rr16SrRNAKhlongMuang [organism= Radix rubiginosa] Radix rubiginosa isolate 16S ribosomal RNA gene, partial sequence  TTTAATAAAGAATTTTCTGTCTTCTTTAAATTTTATTGAATTTATTTAGTAAGTGAAAATACTTAATTAAAGAAAAAAGACGAGAAGACCCTTAGAATTTTTAATAACAACTTTTTTTAAGTTTATTTTTTTGTTGGGGCGACATTTAAACATTTAAACTTTAAATACTCCACTGGACATTAAGTTTTGTGA |
| Snail_WangKaTa | >PP913366 Rr16SrRNAWangKatha [organism= Radix rubiginosa] Radix rubiginosa isolate 16S ribosomal RNA gene, partial sequence  TTTAATAAAGAATTTTCTGTCTTCTTTAAATTTTATTGAATTTATTTAGTAAGTGAAAATACTTAATTAAAGAAAAAAGACGAGAAGACCCTTAGAATTTTTAATAACAACTTTTTTTAAAGTTTATTTTTTTGTTGGGGCGACATTTAAACATTTAAACTTTAAATACTCCACTGGACATTAAGTTTTGTGA |
| Snail_PhayaYen | >PP913367 Rr16SrRNAPhayaYen [organism= Radix rubiginosa] Radix rubiginosa isolate 16S ribosomal RNA gene, partial sequence  TTTAATAAAGAATTTTCTGTCTTCTTTAAATTTTATTGAATTTATTTAGTAAGTGAAAATACTTAATTAGAGAAAAAAGACGAGAAGACCCTTAGAATTTTTAATAACAACTTTTTTTAAGTTTATTTTTTTGTTGGGGCGACATTTAAACATTTAAACTTTAAATACTCCACTGGACATTAAGTTTTGTGA |
| Snail_NongSaRai | >PP913368 Rr16SrRNANongSarai [organism= Radix rubiginosa] Radix rubiginosa isolate 16S ribosomal RNA gene, partial sequence  TTTAATAAAGAATTTTCTGTCTTCTTTAAATTTTATTGAATTTATTTAGTAAGTGAAAATACTTAATTAAAGAAAAAAGACGAGAAGACCCTTAGAATTTTTAATAACAACTTTTTTTAAAGTTTATTTTTTTGTTGGGGCGACATTTAAACATTTAAACTTTAAATACTCCACTGGACATTAAGTTTTGTGA |
| Snail_PongTaLong | >PP913369 Rr16SrRNAPongTalong [organism= Radix rubiginosa] Radix rubiginosa isolate 16S ribosomal RNA gene, partial sequence  TTTAATAAAGAATTTTCTGTCTTCTTTAAATTTTATTGAATTTATTTAGTAAGTGAAAATACTTAATTAGAGAAAAAAGACGAGAAGACCCTTAGAATTTTTAATAACAACTTTTTTTAAGTTTATTTTTTTGTTGGGGCGACATTTAAACATTTAAACTTTAAATACTCCACTGGACATTAAGTTTTGTGA |
|  | >PP913370 Rr16SrRNAKlangDong [organism= Radix rubiginosa] Radix rubiginosa isolate 16S ribosomal RNA gene, partial sequence  TTTAATAAAGAATTTTCTGTCTTCTTTAAATTTTATTGAATTTATTTAGTAAGTGAAAATACTTAATTAAAGAAAAAAGACGAGAAGACCCTTAGAATTTTTAATAATAACTTTTTTAAAGTTTATTTTTTTGTTGGGGCGACATTAAAACATTTAAACTTTAAATACTCCACTGGACATTAAGTTTTGTGA |

**Table S2** All of the *Fasciola* *gigantica* COX1 sequences obtained from our study with their accession numbers.

| **Name code** | **GenBank accession no. and sequence** |
| --- | --- |
| Fg_ChanThuek | >PP907740 FgCOX1Chanthuek [organism= Fasciola gigantica] Fasciola gigantica isolate cytochrome c oxidase subunit I gene partial sequence  TGACGGGGCATGGTGTTATTATGATTTTTTTCTTTTTGATGCCTGTGTTGATTGGGGGGTTTGGTAATTATTTATTGCCTTTGCTTTTGGGTATTCCTGATTTGAATTTGCCTCGTTTAAATGCTTTGAGTGCTTGGTTGTTGCTTCCTGCTTGTGTTTGTTTGTCGTTTGGTTTGATGGGGGGTATGGGTGTTGGTTGGACTTTCTATCCTCCTCTTTCTAGATTGGATTATTCTGGTTGGGGAGTTGATTTTTTAATGTTTTCCCTTCATTTGGCTGGTGTTTCTAGTCTTTTGGGTTCTATTAAATTTATTTGTATTATTTTGGAGGTTATGGTGGGCGAGGGTACTG |
| Fg_MooSi | > PP907741 FgCOX1MuSi [organism= Fasciola gigantica] Fasciola gigantica isolate cytochrome c oxidase subunit I gene partial sequence  TGACGGGGCATGGTGTTATTATGATTTTTTTCTTTTTGATGCCTGTGTTGATTGGGGGGTTTGGTAATTATTTATTGCCTTTGCTTTTGGGTATTCCTGATTTGAATTTGCCTCGTTTAAATGCTTTGAGTGCTTGGTTGTTGCTTCCTGCTTGTGTTTGTTTGTCGTTTGGTTTGATGGGGGGTATGGGTGTTGGTTGGACTTTCTATCCTCCTCTTTCTAGATTGGATTATTCTGGTTGGGGAGTTGATTTTTTAATGTTTTCCCTTCATTTGGCTGGTGTTTCTAGTCTTTTGGGTTCTATTAAATTTATTTGTACTATTTTGGAGGTTATGTTGGGCGAGGGTACTG |
| Fg_PakChong | >PP907742 FgCOX1PakChong [organism= Fasciola gigantica] Fasciola gigantica isolate cytochrome c oxidase subunit I gene partial sequence  TGACGGGGCATGGTGTTATTATGATTTTTTTCTTTTTGATGCCTGTGTTGATTGGGGGGTTTGGTAATTATTTATTGCCTTTGCTTTTGGGTATTCCTGATTTGAATTTGCCTCGTTTAAATGCTTTGAGTGCTTGGTTGTTGCTTCCTGCTTGTGTTTGTTTGTCGTTTGGTTTGATGGGGGGTATGGGTGTTGGTTGGACTTTCTATCCTCCTCTTTCTAGATTGGATTATTCTGGTTGGGGAGTTGATTTTTTAATGTTTTCCCTTCATTTGGCTGGTGTTTCTAGTCTTTTGGGTTCTATTAAATTTATTTGTACTATTTTGGAGGTTATGTTGGGCGAGGGTACTG |
| Fg_KhanongPra | >PP907743 FgCOX1KhanongPhra [organism= Fasciola gigantica] Fasciola gigantica isolate cytochrome c oxidase subunit I gene partial sequence  TGACGGGGCATGGTGTTATTATGATTTTTTTCTTTTTGATGCCTGTGTTGATTGGGGGGTTTGGTAATTATTTATTGCCTTTGCTTTTGGGTATTCCTGATTTGAATTTGCCTCGTTTAAATGCTTTGAGTGCTTGGTTGTTGCTTCCTGCTTGTGTTTGTTTGTCGTTTGGTTTGATGGGGGGTATGGGTGTTGGTTGGACTTTCTATCCTCCTCTTTCTAGATTGGATTATTCTGGTTGGGGGGTTGATTTTTTAATGTTTTCCCTTCATTTGGCTGGTGTTTCTAGTCTTTTGGGTTCTATTAAATTTATTTGTACTATTTTGGAGGTTATGTTGGGCGAGGGTACTG |
| Fg_NongNamDaeng | >PP907744 FgCOX1NongNamDaeng [organism= Fasciola gigantica] Fasciola gigantica isolate cytochrome c oxidase subunit I gene partial sequence  TGACGGGGCATGGTGTTATTATGATTTTTTTCTTTTTGATGCCTGTGTTGATTGGGGGGTTTGGTAATTATTTATTGCCTTTGCTTTTGGGTATTCCTGATTTGAATTTGCCTCGTTTAAATGCTTTGAGTGCTTGGTTGTTGCTTCCTGCTTGTGTTTGTTTGTCGTTTGGTTTGATGGGGGGTATGGGTGTTGGTTGGACTTTCTATCCTCCTCTTTCTAGATTGGATTATTCTGGTTGGGGGGTTGATTTTTTAATGTTTTCCCTTCATTTGGCTGGTGTTTCTAGTCTTTTGGGTTCTATTAAATTTATTTGTACTATTTTGGAGGTTATGTTGGGCGAGGGTACTG |
| Fg_WangSai | >PP907745 FgCOX1WangSai [organism= Fasciola gigantica] Fasciola gigantica isolate cytochrome c oxidase subunit I gene partial sequence  TGACGGGGCATGGTGTTATTATGATTTTTTTCTTTTTGATGCCTGTGTTGATTGGGGGGTTTGGTAATTATTTATTGCCTTTGCTTTTGGGTATTCCTGATTTGAATTTGCCTCGTTTAAATGCTTTGAGTGCTTGGTTGTTGCTTCCTGCTTGTGTTTGTTTGTCGTTTGGTTTGATGGGGGGTATGGGTGTTGGTTGGACTTTCTATCCTCCTCTTTCTAGATTGGATTATTCTGGTTGGGGAGTTGATTTTTTAATGTTTTCCCTTCATTTGGCTGGTGTTTCTAGTCTTTTGGGTTCTATTAAATTTATTTGTACTATTTTGGAGGTTATGTTGGGCGAGGGTACTG |
| Fg_KhlongMuang | >PP907746 FgCOX1KhlongMuang [organism= Fasciola gigantica] Fasciola gigantica isolate cytochrome c oxidase subunit I gene partial sequence  TGACGGGGCATGGTGTTATTATGATTTTTTTCTTTTTGATGCCTGTGTTGATTGGGGGGTTTGGTAATTATTTATTGCCTTTGCTTTTGGGTATTCCTGATTTGAATTTGCCTCGTTTAAATGCTTTGAGTGCTTGGTTGTTGCTTCCTGCTTGTGTTTGTTTGTCGTTTGGTTTGATGGGGGGTATGGGTGTTGGTTGGACTTTCTATCCTCCTCTTTCTAGATTGGATTATTCTGGTTGGGGAGTTGATTTTTTAATGTTTTCTCTTCATTTGGCTGGTGTTTCTAGTCTTTTGGGTTCTATTAAATTTATTTGTACTATTTTGGAGGTTATGTTGGGCGAGGGTACTG |
| Fg_WangKaTa | >PP907747 FgCOX1WangKatha [organism= Fasciola gigantica] Fasciola gigantica isolate cytochrome c oxidase subunit I gene partial sequence  TGACGGGGCATGGTGTTATTATGATTTTTTTCTTTTTGATGCCTGTGTTGATTGGGGGGTTTGGTAATTATTTATTGCCTTTGCTTTTGGGTATTCCTGATTTGAATTTGCCTCGTTTAAATGCTTTGAGTGCTTGGTTGTTGCTTCCTGCTTGTGTTTGTTTGTCGTTTGGTTTGATGGGGGGTATGGGTGTTGGTTGGACTTTCTATCCTCCTCTTTCTAGATTGGATTATTCTGGTTGGGGAGTTGATTTTTTAATGTTTTCCCTTCATTTGGCTGGTGTTTCTAGTCTTTTGGGTTCTATTAAATTTATTTGTACTATTTTGGAGGTTATGTTGGGCGAGGGTACTG |
| Fg_PhayaYen | >PP907748 FgCOX1PhayaYen [organism= Fasciola gigantica] Fasciola gigantica isolate cytochrome c oxidase subunit I gene partial sequence  TGACGGGGCATGGTGTTATTATGATTTTTTTCTTTTTGATGCCTGTGTTGATTGGGGGGTTTGGTAATTATTTATTGCCTTTGCTTTTGGGTATTCCTGATTTGAATTTGCCTCGTTTAAATGCTTTGAGTGCTTGGTTGTTGCTTCCTGCTTGTGTTTGTTTGTCGTTTGGTTTGATGGGGGGTATGGGTGTTGGTTGGACTTTCTATCCTCCTCTTTCTAGATTGGATTATTCTGGTTGGGGGGTTGATTTTTTAATGTTTTCCCTTCATTTGGCTGGTGTTTCTAGTCTTTTGGGTTCTATTAAATTTATTTGTACTATTTTGGAGGTTATGTTGGGCGAGGGTACTG |
| Fg_NongSaRai | >PP907749 FgCOX1NongSarai [organism= Fasciola gigantica] Fasciola gigantica isolate cytochrome c oxidase subunit I gene partial sequence  TGACGGGGCATGGTGTTATTATGATTTTTTTCTTTTTGATGCCTGTGTTGATTGGGGGGTTTGGTAATTATTTATTGCCTTTGCTTTTGGGTATTCCTGATTTGAATTTGCCTCGTTTAAATGCTTTGAGTGCTTGGTTGTTGCTTCCTGCTTGTGTTTGTTTGTCGTTTGGTTTGATGGGGGGTATGGGTGTTGGTTGGACTTTCTATCCTCCTCTTTCTAGATTGGATTATTCTGGTTGGGGGGTTGATTTTTTAATGTTTTCCCTTCATTTGGCTGGTGTTTCTAGTCTTTTGGGTTCTATTAAATTTATTTGTATTATTTTGGAGGTTATGGTGGGCGAGGGTACTG |
| Fg_PongTaLong | >PP907750 FgCOX1PongTalong [organism= Fasciola gigantica] Fasciola gigantica isolate cytochrome c oxidase subunit I gene partial sequence  TGACGGGGCATGGTGTTATTATGATTTTTTTCTTTTTGATGCCTGTGTTGATTGGGGGGTTTGGTAATTATTTATTGCCTTTGCTTTTGGGTATTCCTGATTTGAATTTGCCTCGTTTAAATGCTTTGAGTGCTTGGTTGTTGCTTCCTGCTTGTGTTTGTTTGTCGTTTGGTTTGATGGGGGGTATGGGTGTTGGTTGGACTTTCTATCCTCCTCTTTCTAGATTGGATTATTCTGGTTGGGGGGTTGATTTTTTAATGTTTTCCCTTCATTTGGCTGGTGTTTCTAGTCTTTTGGGTTCTATTAAATTTATTTGTACTATTTTGGAGGTTATGTTGGGCGAGGGTACTG |
| Fg_KlangDong | >PP907751 FgCOX1KlangDong [organism= Fasciola gigantica] Fasciola gigantica isolate cytochrome c oxidase subunit I gene partial sequence  TGACGGGGCATGGTGTTATTATGATTTTTTTCTTTTTGATGCCTGTGTTGATTGGGGGGTTTGGTAATTATTTATTGCCTTTGCTTTTGGGTATTCCTGATTTGAATTTGCCTCGTTTAAATGCTTTGAGTGCTTGGTTGTTGCTTCCTGCTTGTGTTTGTTTGTCGTTTGGTTTGATGGGGGGTATGGGTGTTGGTTGGACTTTCTATCCTCCTCTTTCTAGATTGGATTATTCTGGTTGGGGGGTTGATTTTTTAATGTTTTCCCTTCATTTGGCTGGTGTTTCTAGTCTTTTGGGTTCTATTAAATTTATTTGTACTATTTTGGAGGTTATGTTGGGCGAGGGTACTG |
