# Supplementary material for: Investigation of Fasciola gigantica in freshwater snail Radix (Lymnaea) spp. In the highly parasite-prevalent area of Nakhon Ratchasima Province, Thailand
Source: Int J Vet Sci Med. 2024 Sep 10;12(1):125–33. doi: 10.1080/23144599.2024.2396700 (PMC11389629; doi:10.1080/23144599.2024.2396700)
Supplement: Supplementary Figures revised 2.docx [file TVSM_A_2396700_SM2852.docx]

**Supplementary figure legends**

**Figure S1:** 1% Agarose gel electrophoresis of PCR products amplified from snail gDNA using *Radix rubiginosa* 16S rDNA-specific primers. Lane M is a DNA ladder (GeneRuler 100 bp Plus DNA ladder, Thermo Fisher Scientific, Wilmington, MC, USA). Lanes 1-12 represent the snails collected from each subdistrict, including Chan Thuek, Moo Si, Pak Chong, Khanong Pra, Nong Nam Daeng, Wang Sai, Khlong Muang, Wang Ka Ta, Phaya Yen, Nong Sa Rai, Pong Ta Long, and Klang Dong, respectively. Lane N is negative control.

**Figure S2:** 1% Agarose gel electrophoresis of PCR products from *Fasiola gigantica* COX1-specific primer (upper panel) was amplified using snail gDNA (lower panel). Lane M is a DNA ladder (GeneRuler 100 bp Plus DNA ladder, Thermo Fisher Scientific, Wilmington, MC, USA). Lanes 1-12 represent the snails collected from each subdistrict, including Chan Thuek, Moo Si, Pak Chong, Khanong Pra, Nong Nam Daeng, Wang Sai, Khlong Muang, Wang Ka Ta, Phaya Yen, Nong Sa Rai, Pong Ta Long, and Klang Dong, respectively. Lane N is negative control.


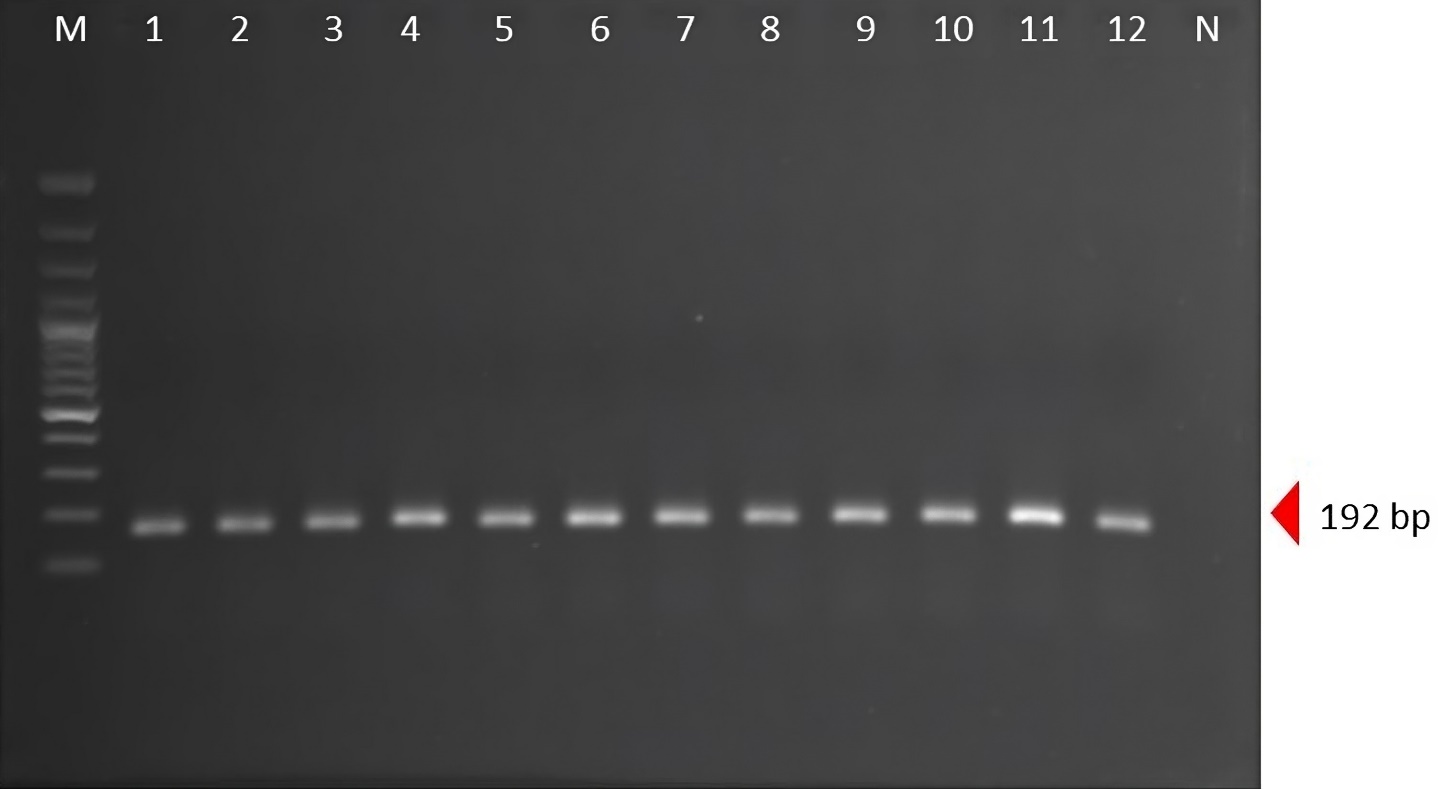
**Figure S1**


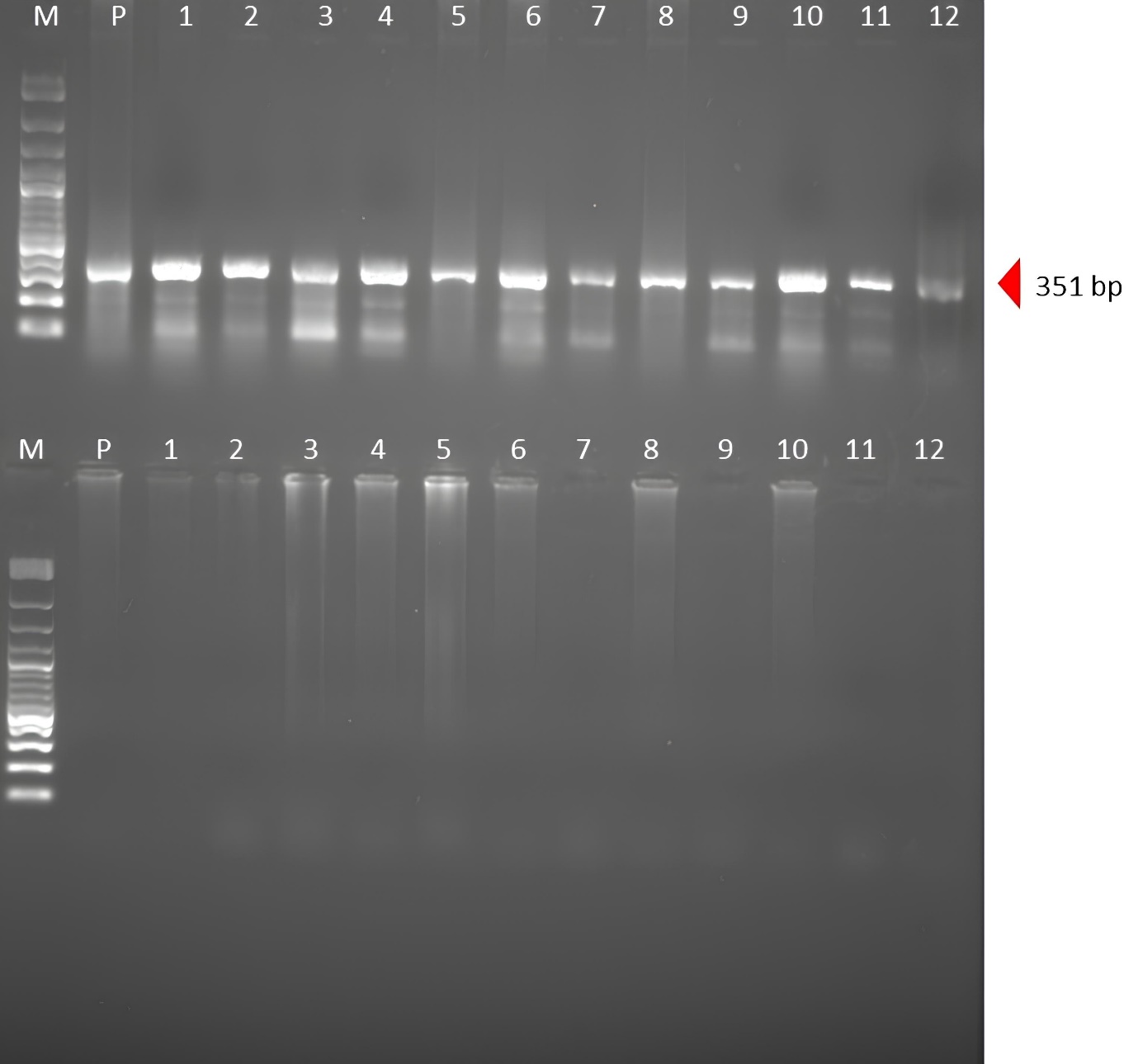
**Figure S2**
